# Response to letter to the editor regarding ‘does point-of care ultrasound examination by the general practitioner lead to inappropriate care? A follow up study’ by Andersen et al

**DOI:** 10.1080/02813432.2025.2516383

**Published:** 2025-06-09

**Authors:** Camilla Aakjær Andersen, John Brandt Brodersen, Martin Bach Jensen

**Affiliations:** a Center for General Practice at Aalborg University, Aalborg University, Aalborg, Denmark; b Centre of General Practice, Department of Public Health, Faculty of Health Sciences, University of Copenhagen, Copenhagen, Denmark; c Research Unit for General Practice, Region Zealand, Denmark; d Research Unit for General Practice, Department of Community Medicine, Faculty of Health Sciences, UiT The Arctic University of Norway, Tromsø, Norway

Dear Editor

We appreciate the opportunity to respond to the letter addressing our paper, ‘does point-of care ultrasound examination by the general practitioner lead to inappropriate care? A follow up study’ published in Scandinavian Journal of Primary Healthcare [1]. We welcome scholarly discourse, as it contributes to refining and advancing our understanding of best clinical practice. In responding to the letter, we would like to highlight the difference between screening and performing point-of-care ultrasound (POCUS) examinations on symptomatic patients and how a distinction between the two played a part in our analysis.

## A distinction between screening and scanning patients with symptoms

In the letter Zachary Boivin (ZB) advice ‘*I encourage the authors to reconsider their approach to using POCUS in early pregnancy to confirm intrauterine pregnancies and provide accurate fetal dating, and allow patients to use the knowledge gained by POCUS to make their own informed decisions through shared decision making*’. We would like to point to a distinction between prenatal screening and performing POCUS on patients with symptoms. In Denmark, all pregnant women are invited to a prenatal screening around week 12 in the pregnancy. The prenatal screening is performed at the hospital and includes a comprehensive ultrasound examination with the aim of making a risk assessment for foetal malformations, establishing gestational age, finding twin pregnancies etc. Ultrasound examinations before week 12 are only performed in cases of unknown gestational age or symptoms such as pain or bleeding. In pregnant asymptomatic women ectopic pregnancy or molar pregnancy are rare. Hence, routine screening with ultrasound for ectopic pregnancy prior to the prenatal screening mentioned above is not recommended [2].

We agree with ZB that the primary goal of POCUS is to answer a clinical question. Medical screening inevitably comes with the risk of false positives, incidental findings and overdiagnosis and this risk of unintended harm is greater when screening asymptomatic people than in symptomatic patients [3] because of the low pre-test probability of abnormalities, spectrum and overdiagnosis bias. Thus, any additional scans initiated at the GP’s office should have a clinical rationale, which we did not find in this case.

Prior to prenatal screening, information about pros and cons as well as possible outcomes of the screening test, is given to the person upon invitation so they can prepare for the examination and make an informed choice whether to participate in the screening or not. Informed consent before an examination based on a clinical indication is given in the consultation with less time for reflection. Still, once the examination is performed, we strongly agree with ZB in his statement that we should allow patients to use the knowledge gained by POCUS to make their own informed decisions through shared decision-making.
