# Letter to the editor regarding ‘does point-of-care ultrasound examination by the general practitioner lead to inappropriate care? A follow-up study’

**DOI:** 10.1080/02813432.2025.2499172

**Published:** 2025-04-28

**Authors:** Zachary Boivin

**Affiliations:** Department of Emergency Medicine, Yale School of Medicine, New Haven, CT, USA

I read with interest ‘Does point-of-care ultrasound examination by the general practitioner lead to inappropriate care? A follow-up study’ by Andersen et al. in the Journal, which found that point-of-care ultrasound (POCUS) was generally safe but had a 5.3% rate of possible misdiagnosis, 0.7% rate of potential overdiagnosis, and 0.7% rate of incidental findings [1]. I agree with their overall findings that POCUS is generally safe, and support the expansion of POCUS into general practice for its bedside diagnostic capability. They mentioned a specific case where a patient had an early pregnancy POCUS at seven weeks, and when a twin gestation was discovered, elected to have an abortion. I would like to address a couple of points brought up in the discussion. They asserted that there was no medical indication for this POCUS examination. While the patient may not have had any symptoms such as vaginal bleeding or abdominal pain, where most of the research into pelvic POCUS centers around, any pregnancy without a confirmed intrauterine pregnancy is a potential ectopic pregnancy [2]. POCUS may provide reassurance to the patient and general practitioner, or uncover an unexpected diagnosis such as ectopic pregnancy or molar pregnancy. At seven weeks of gestation, a yolk sac within a gestational sac can often be visualized in the uterus, confirming a correctly located pregnancy. Additionally, a patient’s estimated last menstrual period can be incorrect, resulting in improper prenatal care if the fetus is not accurately dated [3]. The authors also state that ‘had the twin pregnancy been identified during prenatal scanning, [the patient] might not have chosen an abortion’. While the authors ultimately were unable to classify this re-consultation as related to the POCUS, the discussion insinuated a decision was made immediately after the POCUS (‘after receiving this result, the woman decided to have an abortion’), while their supplemental file reports the decision was made over multiple consultations and was a well-thought out and informed decision by the patient, albeit without the involvement of obstetrics and gynecology. The primary goal of POCUS is to answer a clinical question, in this case whether there was an intrauterine pregnancy or not, and I believe the practitioner used POCUS appropriately in this case. As this study showed, POCUS rarely leads to misdiagnosis or overdiagnosis, and other studies show protection against medical malpractice through its ability to diagnose conditions at the bedside [4,5]. I encourage the authors to reconsider their approach to using POCUS in early pregnancy to confirm intrauterine pregnancies and provide accurate fetal dating, and allow patients to use the knowledge gained by POCUS to make their own informed decisions through shared decision making.
